# HGF Overexpression in Mesenchymal Stromal Cell-Based Cell Sheets Enhances Autophagy-Dependent Cytoprotection and Proliferation to Guard the Epicardial Mesothelium

**DOI:** 10.3390/ijms26157298

**Published:** 2025-07-28

**Authors:** Konstantin Dergilev, Irina Beloglazova, Zoya Tsokolaeva, Ekaterina Azimova, Aleria Dolgodvorova, Yulia Goltseva, Maria Boldyreva, Mikhail Menshikov, Dmitry Penkov, Yelena Parfyonova

**Affiliations:** 1 Institute of Experimental Cardiology Named after Academician V.N. Smirnov, Federal State Budgetary Institution National Medical Research Center of Cardiology Named after Academician E.I. Chazov, Ministry of Health of the Russian Federation, 121552 Moscow, Russiaaitova.aa@phystech.edu (A.D.);; 2 Federal Research and Clinical Center of Intensive Care Medicine and Rehabilitology, Solnechnogorsk District, v. Lytkino, 141534 Moscow, Russia; 3 IFOM (Foundation FIRC Institute of Molecular Oncology), Via Adamello 16, 20139 Milan, Italy

**Keywords:** epicardial cells, HGF, cell sheets, myocardial infarction

## Abstract

Epicardial mesothelial cells (EMCs), which form the epicardium, play a crucial role in cardiac homeostasis and repair. Upon damage, EMCs reactivate embryonic development programs, contributing to wound healing, progenitor cell amplification, and regulation of lymphangiogenesis, angiogenesis, and fibrosis. However, the mechanisms governing EMC activation and subsequent regulation remain poorly understood. We hypothesized that hepatocyte growth factor (HGF), a pleiotropic regulator of various cellular functions, could modulate EMC activity. To verify this hypothesis, we developed HGF-overexpressing mesenchymal stromal cell sheets (HGF-MSC CSs) and evaluated their effects on EMCs in vitro and in vivo. This study has revealed, for the first time, that EMCs express the c-Met (HGF receptor) on their surface and that both recombinant HGF and HGF-MSC CSs secretome cause c-Met phosphorylation, triggering downstream intracellular signaling. Our findings demonstrate that the HGF-MSC CSs secretome promotes cell survival under hypoxic conditions by modulating the level of autophagy. At the same time, HGF-MSC CSs stimulate EMC proliferation, promoting their amplification in the damage zone. These data demonstrate that HGF-MSC CSs can be considered a promising regulator of epicardial cell activity involved in heart repair after ischemic damage.

## 1. Introduction

The increasing global prevalence of myocardial infarction is a primary driver of morbidity and mortality worldwide [1]. Recent advancements have significantly improved our understanding of myocardial infarction pathogenesis [2], revealing the critical role of the epicardium in orchestrating the regenerative response. The epicardium acts as a signaling hub, influencing diverse cellular activities within the heart [3,4]. Traditionally viewed as a quiescent protective layer, the epicardium is now recognized as a dynamic participant in cardiac development, repair, and regeneration [5]. It is vital in supplying signaling factors, progenitor cells, and paracrine signals that are essential for regulating inflammation, promoting the proliferation of progenitor cells and cardiomyocytes, and controlling lymphogenesis, angiogenesis, and fibrosis. Additionally, recent studies suggest that reactivating the epicardium may unlock its dormant regenerative capacity, offering new therapeutic avenues to enhance cardiac repair and improve outcomes following myocardial infarction (MI). Acute ischemic injury is characterized by the loss of EMCs, disruption of the basement membrane, overproliferation of fibroblasts, and excessive accumulation of the extracellular matrix [6]. While the pathogenesis of epicardial damage remains to be elucidated, mesothelial cell depletion is thought to be a critical factor contributing to both increased damage and inhibited repair. Therefore, further investigation into the mechanisms behind EMC damage and the factors regulating their state and activation, as well as their impact on cardiac regeneration, is essential for developing effective interventions against post-infarction cell death and heart failure. Numerous studies have focused on targeting the epicardium and creating an environment suited for epicardial layer repair by employing appropriate regulatory signals, progenitor cells, extracellular matrix (ECM), or supportive constructs [3,4]. One promising approach to epicardial repair is cell sheet technology, which utilizes cultured cells and their secreted ECM to create a microenvironment optimal for tissue regeneration. This method allows a specialized microenvironment to be generated that enhances cell survival, activity rates, and favorable cell–ECM interactions.

Given the aforementioned findings and our earlier results, we posit that the efficacy of MSCs against EMCs can be improved by creating an MSC-based cell sheet (CS) engineered for HGF overproduction. HGF was first identified as a factor that promotes epithelial cell motility [7], and its properties were later expanded to include the regulation of apoptosis, proliferation, and morphogenesis in various epithelial tissues. Recent studies have demonstrated the involvement of the receptor encoded by the proto-oncogene MET in essential reparative functions [8], including the implementation of pro-inflammatory and immunomodulatory signals, cardioprotection, stimulation of angiogenesis, and the suppression of fibrosis [9].

This study is the first to demonstrate that epicardial cells express the c-Met receptor on their surface. We found that exposure to recombinant HGF and the HGF-MSC CSs secretome induces its phosphorylation and triggers intracellular signaling. In addition, the secreted products of control and HGF-producing cells within the construct were found to ensure cell survival under hypoxic conditions. Additionally, HGF-MSC CSs were observed to induce enhanced EMC proliferation. In both in vivo and in vitro models, HGF-MSC CSs have been demonstrated to modulate autophagy levels, facilitating the restoration of the EMC population within damaged areas. Our findings strongly suggest that HGF-MSC CS has the potential to function as a promising regulator of the EMC activity involved in cardiac repair following ischemic injury.

## 2. Results

### 2.1. Genetically Modified MSCs Form Stable Cell Sheets and Are Characterized by a Prolonged Capacity to Produce HGF

**Figure 1:**
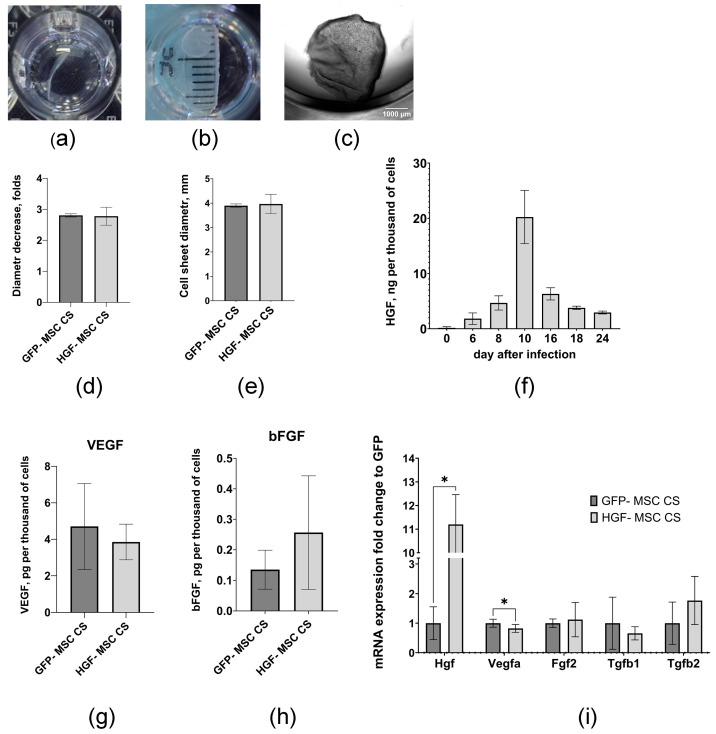
The characteristics of the CSs. The 72 h cell sheets were washed three times with Versene solution and then incubated in Versene solution for 3–5 min at 37 °C and 5% CO_2_. The representative images of the CSs: (**a**) partly detached CSs (brightfield image), (**b**) completely detached CSs (brightfield image), (**c**) completely detached CSs (phase-contrast image). The diameters of CSs from (**c**) were analyzed using the ImageJ software (v 1.54d, NIH, USA). (**d**) The average diameters of the detached GFP-MSC CSs and HGF-MSC CSs. (**e**) The average folds of the GFP-MSC CS and HGF-MSC CS contraction after detachment. (**f**) HGF production by HGF-MSC for 24 days evaluated by ELISA. (**g**) VEGF secretion by GFP-MSC CSs and HGF-MSC CSs for 48 h on day 8 after infection, as evaluated by ELISA. (**h**) bFGF secretion by GFP-MSC CSs and HGF-MSC CSs for 48 h on day 8 after infection evaluated by ELISA. (**i**) Analysis of growth factor genes. Total RNA of GFP-MSC and HGF-MSC was isolated on day 11 after infection, and specific mRNA levels were quantified by qRT PCR, as described in the Section 4. The data are presented as fold changes in the mRNA levels normalized to GFP-MSC “*”: *p* < 0.05, *n* ≥ 4.

**Figure 2:**
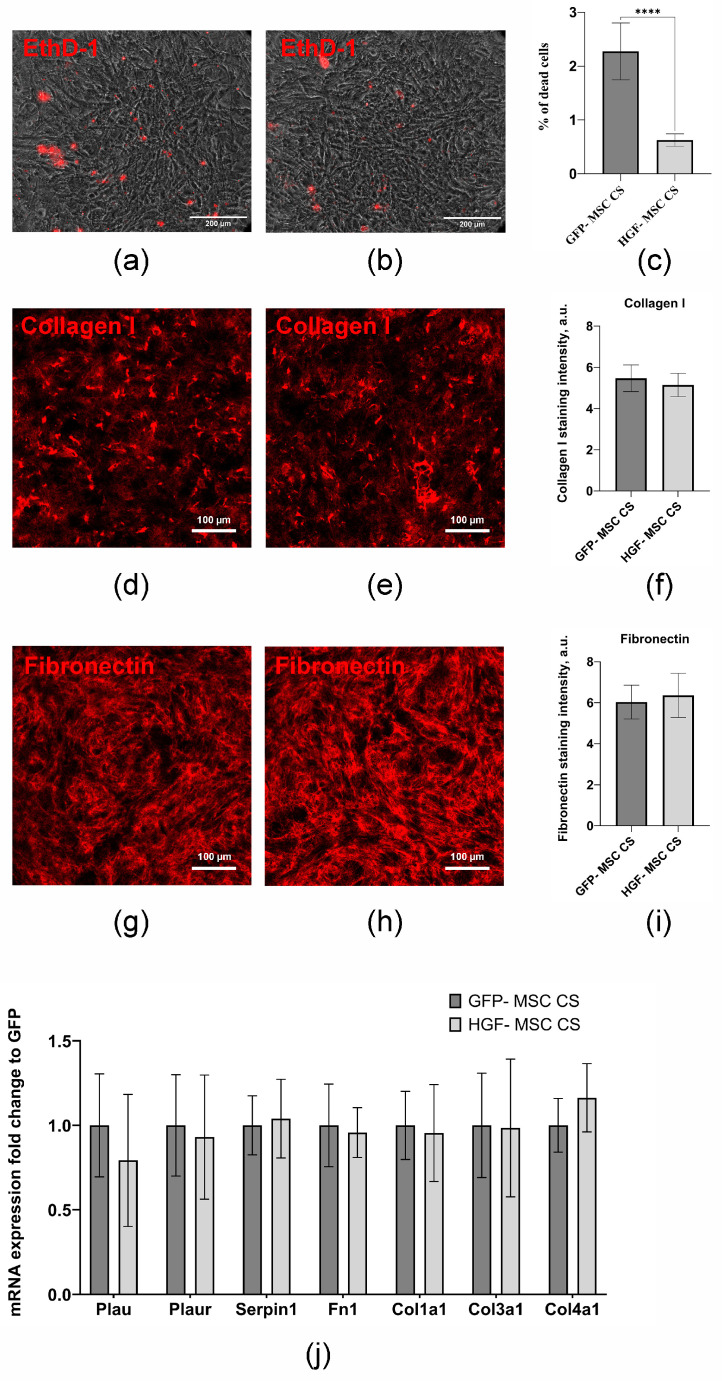
Characteristics of CSs, whereby 72 h CSs were stained with LIVE/DEAD™ Viability/Cytotoxicity Kit. (**a**,**b**) % of dead cells in CS. Representative fluorescence images of CSs stained with ethidium homodimer-1: (**a**) GFP-MSC CSs and (**b**) HGF-MSC CSs. The percentage of dead cells was analyzed using ImageJ software (v 1.54d, NIH, USA) (**c**) Quantification of the percentage of the dead cells for GFP-MSC CSs and HGF-MSC CSs. (**d**–**f**) Analysis of collagen I deposition in the 72 h CSs. (**d**) GFP-MSC CSs and (**e**) HGF-MSC CSs were fixed with 4% formaldehyde, and stained with the anti-collagen I antibody (red). Images were taken using a Stellaris 5 confocal microscope (Leica, Wetzlar, Germany). (**f**) Quantification of the intensities of staining for collagen I using Image J software (v 1.54d, NIH, USA). (**g**–**i**) Analysis of fibronectin deposition in the cell layer. (**g**) GFP-MSC CSs and (**h**) HGF-MSC CSs were fixed with 4% formaldehyde and stained with the anti-collagen I antibody (red). The images were taken using a Stellaris 5 confocal microscope (Leica, Germany). (**i**) Quantification of the intensities of staining for fibronectin using Image J software. (**j**) Analysis of extracellular matrix genes. Total RNA of GFP-MSC and HGF-MSC was isolated on day 11 after infection and specific mRNA levels were quantified by qRT PCR as described in the Section 4. The data are presented as fold changes in the mRNA levels normalized to GFP-MSC “****”: *p* < 0.001, *n* ≥ 4.

Mouse MSCs transduced with recombinant adeno-associated virus encoding the mouse HGF gene or GFP (as control) were used to generate genetically modified cells. We used adeno-associated virus serotype DJ, which was previously found to exhibit high transduction efficiency (over 90%) [10]. The formation of multilayered round-shaped CSs was observed in both cell types, with no significant differences in size. The mean diameter of detached HGF-MSC CSs was 3.97 ± 0.40 mm (*n* = 5), and that of the control MSC CSs was 3.90 ± 0.07 mm (*n* = 5) (Figure 1a–e). The cells in CSs demonstrated high viability rates. The HGF-MSC CSs exhibited reduced levels of apoptosis compared to the GFP-MSC CSs, with the former having 0.63 ± 0.12% (*n* = 5) of dead cells and the latter having 2.27 ± 0.53% (*n* = 6) (Figure 2a–c). These results indirectly suggest the cytoprotective potential of HGF. The HGF-MSC cells were characterized by a high level of HGF secretion (Figure 1f), determined by immunosorbent assay, reached a maximum (20.23 ± 4.82 ng (relative to 1 × 10^3^ cells)) on day 10 after viral transduction and then smoothly decreased by day 24 to 2.94 ± 0.26 ng/mL. At the same time, the modification of the HGF cells did not lead to an increase in VEGF and bFGF secretion levels or the expression of TGFb1 and TGFb2 genes regulating the state of epicardial cells, compared with GFP-MSC CSs (Figure 1g–i).

Structural studies did not reveal any differences between the two types of cell sheets in terms of thickness and expression of key components of the extracellular matrix (ECM), fibronectin and collagens 1, 3 and 4, as well as the components of the urokinase system (*Plau*, *Plaur*, *Serpine1*) involved in the remodeling of the 3D microenvironment (Figure 2d–j) [11,12]. Based on these data, the HGF-MSC CSs and GFP-MSC CSs obtained on day 6 or their secretome obtained on day 9 after viral transduction were used for subsequent experiments.

### 2.2. HGF-MSC CSs Secretome Stimulates c-Met Receptor Phosphorylation and Downstream Signaling in Epicardium Cells

**Figure 3:**
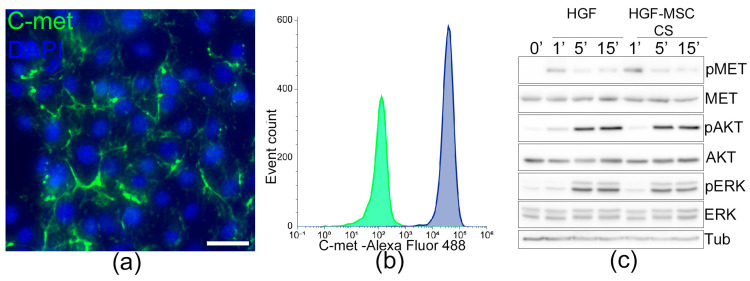
Mouse epicardial cells expressing the c-Met receptor and activating intracellular signaling upon exposure to HGF and HGF-MSC CSs secretome. (**a**) Immunocytochemical analysis of c-Met receptor expression (green) on the surface of the EMCs. The nuclei were counterstained with DAPI. Scale bar: 50 μM. (**b**) The results of the c-Met expression analysis in the EMCs were obtained using flow cytometry. Immunolabelling with c-Met antibodies (blue; control (green)) revealed its presence on the surface of more than 90% of the EMCs. (**c**) Phosphorylation of the c-Met receptor and Akt; ERK signaling pathways after exposure to recombinant HGF and HGF-MSC CSs secretome for 1, 5, and 15 min determined by western blot.

The transplantation of CSs onto the surface of a damaged heart provides both contact and paracrine effects for EMCs, which can be achieved through various factors, including HGF. To investigate the role of HGF in regulating epicardial cell properties, we evaluated the expression of its receptor, c-Met, using flow cytometry and immunoblotting. We found that most EMCs are characterized by the presence of c-Met on the cell surface (Figure 3a,b). Additionally, the secretion of HGF-MSC CSs, combined with exposure to recombinant HGF, induces the phosphorylation of c-Met and triggers the downstream AKT and ERK signaling pathways (Figure 3c).

### 2.3. CSs Transplantation After Infarction Provides Epicardial Cell Recovery and Modulates Autophagy Levels

**Figure 4:**
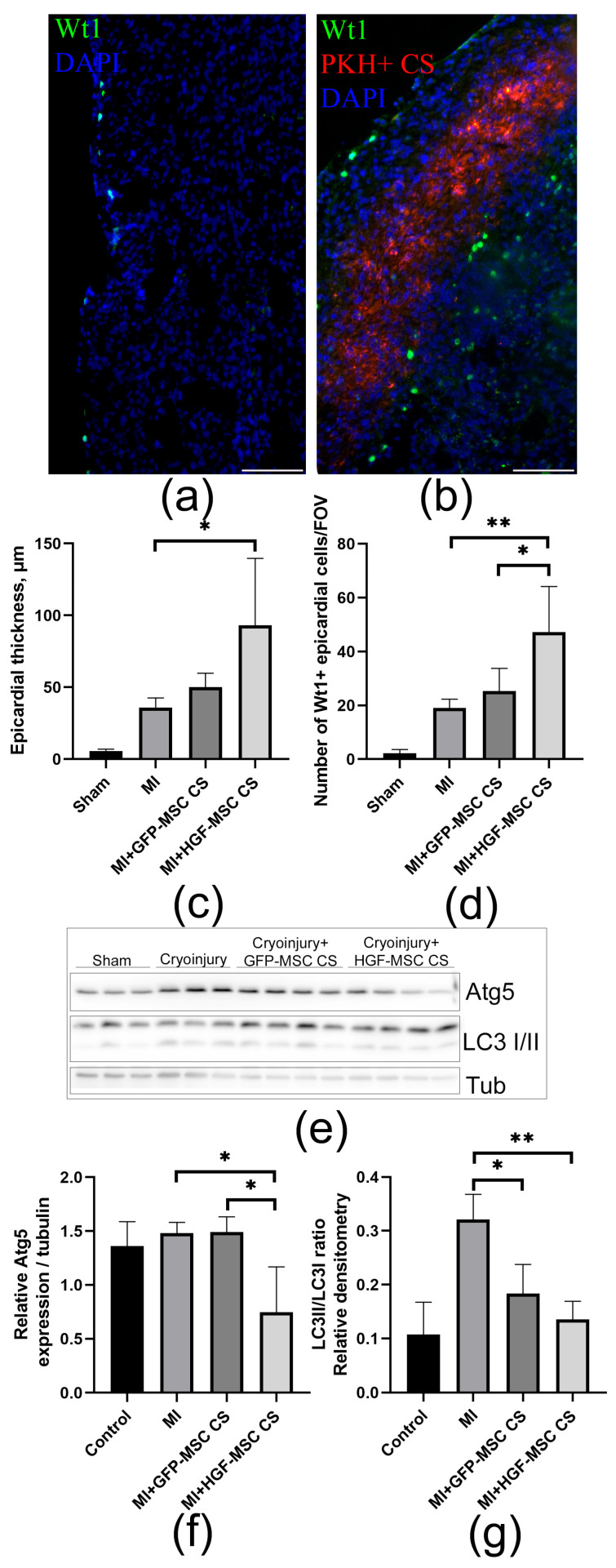
The transplantation of HGF-MSC CSs increases the number of Wt1+ EMCs in the damaged area and regulates the level of autophagy. (**a**,**b**) The representative images of heart tissue after cryoinjury (**a**) and cryoinjury+ HGF-MSC CSs transplantation (**b**) with antibodies against epicardial marker—Wt1 (green). The transplanted HGF-MSC CS (**b**) are labelled with PKH (red). The cell nuclei are stained with DAPI (blue). Scale bar: 50 mkm. (**c**,**d**) The thickness of the epicardial zone (**c**) and the number of Wt1+ EMCs (**d**) in the hearts of sham, cryoinjury, and cell sheet groups (cryoinjury after transplantation of HGF-MSC and GFP-MSC CSs) were calculated and presented in graphs. ANOVA; * *p* < 0.05, ** *p* < 0.01; *n* ≥ 4. (**e**) Atg5 and LC3 I/II expression were analyzed by western blot. The results are shown in the representative images. (**f**,**g**) Densitometric quantification of the Atg5 and LC3 II/I ratio in the epicardial cells of cryoinjured hearts (day 2 after surgery) of the control group and groups, which received a transplant of either GFP- or HGF-MSC CSs. Tubulin was used as loading control. All the data are expressed as mean ± standard deviation (SD) from at least three experiments. * *p* < 0.05, ** *p* < 0.01.

Ensuring EMC viability, proliferation, and subsequent migration into the underlying layers of the heart wall is important for post-infarction repair, and targeting these processes is a promising approach [3,13]. Therefore, after modeling cryoinjury, we investigated the effect of two types of CSs on the epicardial cell layer. After transplantation onto the epicardium, both types of CSs remained viable and integrated into the cardiac tissue. We found that, compared to the control group, HGF-MSC CSs transplantation resulted in an increase in the thickness of the epicardial regions and preservation of EMCs (Figure 4a–c). Interestingly, the effect of the HGF-MSC CSs was significantly more pronounced compared to the GFP-MSC CSs and was accompanied by a more than twofold increase in the number of Wt1+ (Wilms’ tumor gene 1+) cells (Figure 4d).

To evaluate the possible mechanism of the identified changes, we examined the expression of key proteins involved in the autophagy machinery (Atg5 and LC3I/II), a process responsible for the maintenance of cellular homeostasis and the selection between survival and cell death strategies after injury. We found that cryoinjury modeling causes the upregulation of autophagy-related protein expression, while transplanted HGF-MSC CSs reduce the levels of pro-autophagic markers (Figure 4e–g), which may explain the recovery of EMCs in the injury zone.

### 2.4. HGF-MSC CSs Secretome Protects Epicardial Cells from CoCl_2_-Induced Death and Stimulates Their Proliferation

**Figure 5:**
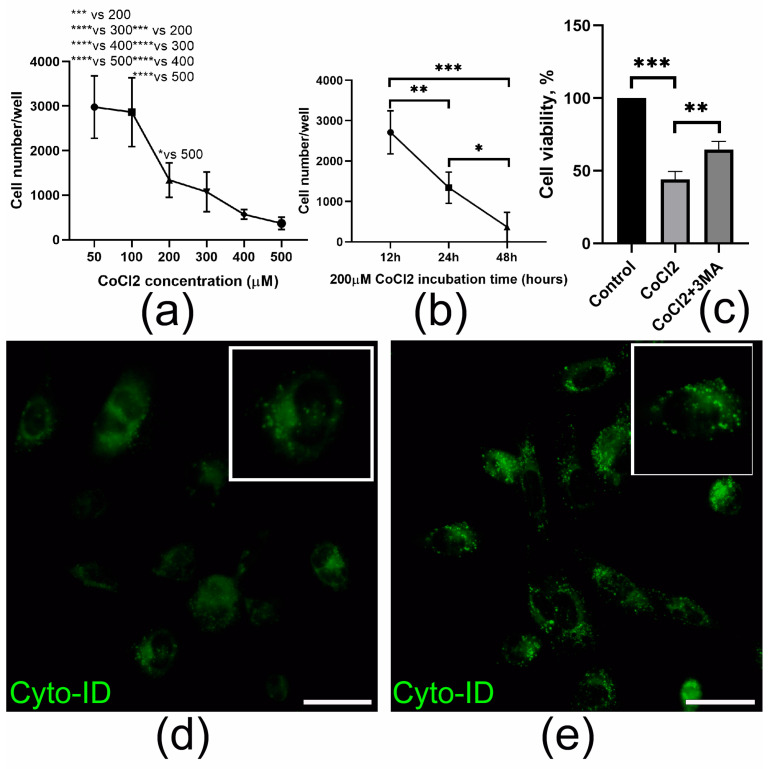
The CoCl_2_ treatment induces autophagosome formation and decreases EMCs survival. (**a**,**b**) Cell viability was analyzed using PrestoBlue assay after exposure to CoCl_2_ at the indicated doses (**a**) and time intervals (**b**). (**c**) Cell viability was analyzed using PrestoBlue assay in control (untreated), cells treated with CoCl_2_ (200 mkM) and CoCl_2_ (200 mkM) + 3MA(10 mkM). (**d**,**e**) Autophagic vacuoles in epicardial cells were visualized using Cyto-ID^®^ in the untreated EMCs and cells after 12 h of exposure to CoCl_2_ (200 μM). Scale bar: 50 mkm. All the data are expressed as mean ± standard deviation (SD) from three or more replications. * *p* < 0.05, ** *p* < 0.01, *** *p* < 0.005 and **** *p* < 0.001.

**Figure 6:**
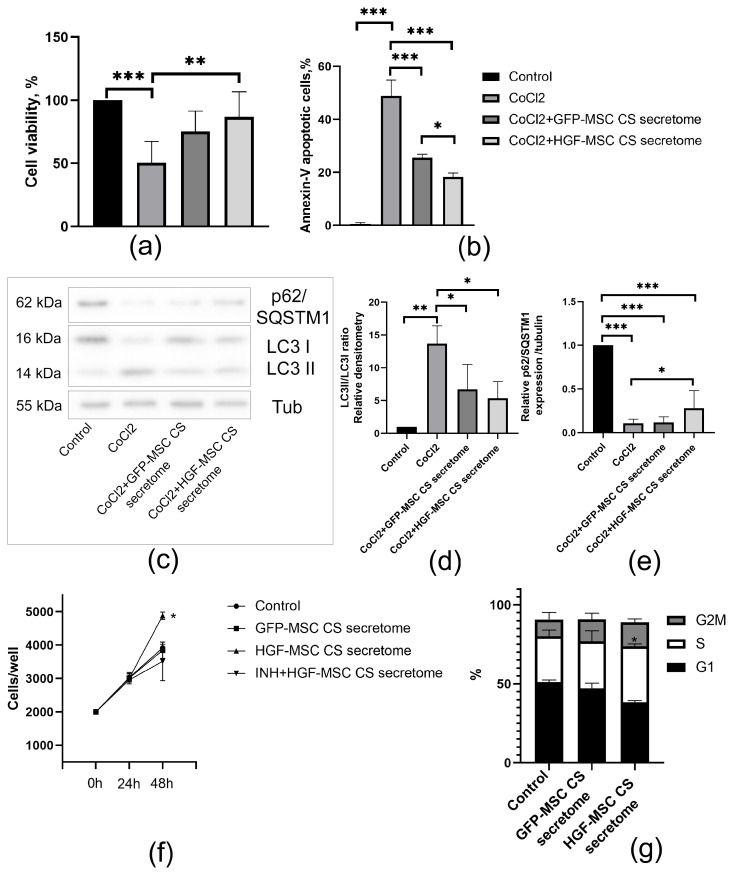
The HGF-MSC CSs secretome protects EMCs from CoCl_2_-induced apoptosis and stimulates their proliferation. (**a**) Cell viability was analyzed using PrestoBlue assay in control (untreated) and cells treated with CoCl_2_ (200 mkM), CoCl_2_ (200 mkM) + GFP-MSC CSs secretome and CoCl_2_ (200 mkM) + HGF-MSC CSs secretome. The results are represented as relative viability (% of untreated control). (**b**) Apoptosis level was analyzed using Annexin-V/PI assay in control (untreated) and cells treated with CoCl_2_ (200 mkM), CoCl_2_ (200 mkM) + GFP-MSC CSs secretome and CoCl_2_ (200 mkM) + HGF-MSC CSs secretome. The results are represented as the presence of Annexin V+ cells relative to control. (**c**) The representative images of western blot analysis demonstrate the p62/SQSTM1 and LC3 I/II expression in the control (untreated) cells and cells treated with CoCl_2_ (200 mkM), CoCl_2_ (200 mkM) + GFP-MSC CSs secretome and CoCl_2_ (200 mkM) + HGF-MSC CSs secretome. (**d**,**e**) Densitometric quantification of the p62/SQSTM1 and LC3 II/I ratio in the control (untreated) and cells treated with CoCl_2_ (200 mkM), CoCl_2_ (200 mkM) + GFP-MSC CSs secretome and CoCl_2_ (200 mkM) + HGF-MSC CSs secretome. Tubulin was used as a loading control. (**f**) Growth curve of control and cells treated for 48 h with GFP-MSC CS and HGF-MSC CS secretomes. To determine the effect of HGF-MSC CSs secretome on cell proliferation, the c-Met inhibitor crizotinib (100 nM) was added to the culture medium. (**g**) The EMCs were incubated for 36 h in control or GFP-MSC CSs and HGF-MSC CSs secretomes. Cell cycle analysis was performed using flow cytometry. All the data are expressed as mean ± standard deviation (SD) from three or more replications. * *p* < 0.05, ** *p* < 0.01, and *** *p* < 0.005.

The induction of acute ischemia results in the death of a significant number of cardiac cells within the first hours after the onset of myocardial infarction [14,15]. To study the cytoprotective effect of the secretome of cell layers on EMCs, we used CoCl_2_, a simple tool for modeling hypoxic exposure in vitro. To investigate the effect of CoCl_2_ on cell viability, we performed the PrestoBlue assay. CoCl_2_ treatment resulted in a significant decrease in epicardial cell survival, which was time- and dose-dependent (Figure 5a,b). Treatment with CoCl_2_ resulted in an elevated number of autophagic vacuoles (Figure 5d,e), as evidenced by the CYTO-ID^®^ assay. Additionally, there was an increase in the LC3II/LC3I ratio and a decrease in p62/SQSTM1 expression, suggesting enhanced autophagy activity in the EMCs (Figure 6c,d). It is important to note that the suppression of autophagy by 3-methyladenine (3MA) induced cell survival in the presence of CoCl_2_, thus highlighting the importance of modulating this process for maintaining cell viability under hypoxic conditions (Figure 5c).

Next, we tested how CSs-conditioned media can affect EMCs survival after CoCl_2_-induced injury. We found that both GFP- and HGF-MSC CSs secretomes could significantly reduce EMC death, as confirmed by PrestoBlue and Annexin-V assays (Figure 6a,b). Furthermore, the number of viable cells following exposure to cobalt salt was found to be significantly higher in the HGF-MSC CSs secretome treatment group compared to the control GFP-MSC CSs group.

Next, we investigated the effect of the secretomes of the GFP-MSC and HGF-MSC CSs on the proliferation activity of EMCs. As demonstrated in Figure 6f, the HGF-MSC CSs secretome promoted the proliferation of epicardial cell cultures, while no effect was observed with the GFP-MSC CSs secretome. It is interesting to note that the addition of a c-Met inhibitor (crizatinib) led to a decrease in the growth rate of EMCs compared to HGF-MSC CSs secretome treatment over 48 h, thereby confirming the involvement of HGF in the regulation of cell division.

A cell cycle assay was performed in order to confirm the proliferative inducer properties of the HGF-MSC CSs secretome. As illustrated in Figure 6g, treatment of Mec-1 with HGF-MSC CSs secretome resulted in a significant increase in the number of cells in the S phase when compared to cells cultured in the base medium. Thus, cell survival under hypoxic conditions and increased proliferation of EMCs was ensured by treatment with HGF-MSC CSs secretome.

## 3. Discussion

The growing body of literature, which is updated annually, underscores the significant role of HGF in facilitating reparative processes in vivo across various organs. Previous studies have demonstrated that HGF exerts multiple beneficial effects, such as reducing inflammation [16,17,18,19], enhancing angiogenesis [20,21,22,23], suppressing fibrosis [24,25,26,27,28,29], activating progenitor cells [30,31,32,33], and providing cytoprotection [34,35]. Despite these promising findings, significant questions remain to be answered regarding the optimal HGF delivery strategies and the identification of specific targets and molecular mechanisms governing its effects. To address these gaps, our laboratory developed a novel approach utilizing cell sheets based on MSCs that exhibit HGF hyperexpression. This method is based on multicellular 3D layers formed by cells and their extracellular matrix proteins. This approach has several distinct advantages over traditional methods, including the injection of cell suspensions or delivery of cells in biodegradable scaffolds [36,37]. These advantages are predicated upon the retention of intercellular and extracellular matrix interactions, which resemble a microenvironment for cells and are necessary for their function and the realization of pro-reparative properties. In a model of lower limb ischemia, HGF-MSC CSs demonstrated good therapeutic results associated with superior restoration of blood flow, tissue vascularization and innervation, and reduced fibrosis [10].

Building on these observations, our study specifically aimed to investigate the potential effect of HGF-MSC CSs on epicardial cells. EMCs, residing within the visceral layer of the pericardium, form a continuous layer of quiescent cells. Once transplanted onto the surface of the heart, cell sheets exert both contact and paracrine interactions with epicardium, which are important for repair. Here, we report for the first time that EMCs express the c-Met receptor on their surface and that exposure to the HGF-MSC CSs secretome causes its phosphorylation and initiation of intracellular signaling (Figure 3). Additionally, the secretory products of control and HGF-producing cells in the construct were found to ensure cell survival under hypoxic conditions (Figure 6a,b). It is worth noting that HGF-MSC CSs further promoted EMC proliferation and modulated autophagy in vivo, increasing cell numbers and accelerating recovery in the damage zone (Figure 4). Therefore, EMCs can be considered a “first-line” target for HGF-producing cell constructs to be delivered to the cardiac surface.

In order to elucidate the mechanisms underlying the HGF-MSC CS**s** effects, we developed a model of heart damage associated with hypoxia. An in vivo myocardial cryoinjury model was used [38,39]. This approach allowed us to control the extent of epicardial damage and its effect on the coronary artery, resulting in ischemia [40,41]. For modeling ischemic damage to EMCs in vitro, a common model of chemical hypoxia using cobalt salts was employed. The development of hypoxia was demonstrated to be accompanied by an increase in the number of autophagic vacuoles observed by CYTO-ID^®^ (Figure 5d,e), an increase in the expression of proteins associated with autophagy (LC3II/LC3I ratio) and a decreased p62/SQSTM1 level (Figure 6c–e). It is hypothesized that, within the heart, autophagy is maintained at a basal level to ensure homeostasis through the processes of cleavage and recycling of misfolded/damaged proteins and organelles.

At the same time, maladaptive changes in autophagy levels can lead to the disruption of regulatory systems and serve as a factor in the development of diseases such as ischemic heart disease and heart failure [42]. When ischemia occurs, associated with the acute disruption of blood supply, adenosine monophosphate-activated protein kinase (AMPK) is activated. AMPK is an energy balance regulator that is activated when ATP levels are reduced, and the AMP/ATP ratio is high under conditions of restricted blood supply and nutrient deprivation [43,44]. Activated AMPK is involved in the activation of autophagy by preventing the MTORC1-dependent inhibition of ULK1 [45,46,47]. Additionally, recent studies have identified new pathways by which AMPK activates autophagy. AMPK directly phosphorylates ULK1 [48,49] and BECN1 [50], thereby initiating the autophagy process.

It should be emphasized that the activation of autophagy under hypoxia is due to different mechanisms, which may exert bidirectional effects on cell survival. Previous research demonstrated that autophagy was considered a protective intracellular process, and its hypoxia-associated activation was shown to exert a cytoprotective function [51,52], including the HIF-1a-dependent activation of vascular endothelial growth factor (VEGF) expression [53] and Bcl-xL [54]. Decker and Wildenthal’s observations indicated that the process of autophagy was associated with the functional recovery of the rabbit heart following ischemia-reperfusion injury [55]. They noted that prolonged ischemia resulted in impaired function of the autophagosome-lysosomal pathway, which was associated with irreversible damage and contractile dysfunction. In a further study, the authors found that inhibition of autophagy in cardiomyocytes with 3MA or Wortmannin, or by expression of the dominant negative ATG5K130R, sensitized cardiac cells to ischemia-mediated cell death, and that enhancement of autophagy was cytoprotective [56]. In other studies, uncontrolled autophagy leads to the excessive degradation of cellular components, the activation of the internal apoptosis pathway involving ROS [57], P53 [58], and Bnip3 [59], and, ultimately, to cell death, known as “autophagic cell death”. Consequently, the maintenance of autophagy activity in the heart may play a dual role. Basal levels of autophagy are necessary for maintaining cellular homeostasis, clearing cellular waste and damaged organelles. Concurrently, a short-term elevation in autophagy during stress has been demonstrated to promote survival, while excessive and prolonged elevations in autophagy have been associated with cell death.

In the present study, CoCl_2_ upregulated autophagic markers and induced epicardial cell death (Figure 5a,b,d,e). These results are consistent with previous reports in cardiomyocytes and endothelial cells. At the same time, the use of CoCl_2_ and the autophagy blocker 3MA, which inhibits PI3K, resulted in a reduction in cell death. After transplantation of both types of CS, the expression of autophagy-regulating proteins tended to decrease in damaged heart tissue, but only for HGF-MSC CSs were these data statistically significant (Figure 4e–g). Although autophagy performs useful regulatory functions in maintaining cardiac cell homeostasis, increased induction of autophagic flux during ischemia is maladaptive and leads to cell death [60,61].

A probable explanation for the decrease in autophagy levels to threshold values may be the effect of the HGF-MSC CSs secretome. This may be based on the effect of HGF’s interaction with the receptor (c-Met), which leads to its dimerization and autophosphorylation, activating phosphoinositide-3-kinase (PI3K)-protein kinase B (AKT), which is closely associated with the intracellular regulation of autophagy [62,63]. AKT has been implicated in the direct activation of MTORC1 by phosphorylation at Ser2448 [64], and indirect activation through the phosphorylation of the GTPase-activating protein TSC2, leading to the suppression of autophagy [65]. Another potential mechanism through which AKT may regulate autophagy levels is the phosphorylation of BECN1 at Ser295, as well as ULK1 at Ser774, critical regulators involved in autophagy initiation [66,67]. Of particular interest is the fact that c-met contains binding sites for LC3, which may also influence the mechanism of autophagosome assembly [68].

It is worth noting that the GFP-MSC CSs has been observed to enhance EMC survival and reduce autophagy flux (Figure 6a,b). This phenomenon may be attributed to the activation of the AKT-mTOR axis in response to secretome components. Combining optimal autophagy level control with the HGF-MSC CSs secretome influence on downstream signaling pathways through the regulation of p21 and p27, cyclin D1, and CDK2 may also contribute to enhancing the survival and proliferation of epicardial cells [69,70,71]. The simultaneous inhibition of c-Met/HGF signaling with crizatinib caused a slight decrease in epicardial cell proliferation stimulated by the secretome, suggesting the multifaceted actions of secretome regulatory proteins (Figure 6f). Consequently, HGF can act in synergy with other growth factors to regulate epithelial proliferation. For instance, in mammary epithelial cells, HGF and EGF have been observed to interact while enhancing proliferation, migration, and invasion. The aforementioned interaction involves the activation of ERK1/2 and AKT, with ERK1/2 inhibition shown to abolish the cooperative effects of HGF and EGF [72]. Of significance is the potential for intracellular interaction between the HGF/c-Met axis and other types of tyrosine kinases. This interaction can result in the activation of various downstream signaling pathways, including, but not limited to, PI3K/AKT, JAK/STAT, Ras/MAPK, SRC, and Wnt/β-catenin [71,73,74,75,76].

This study has potential limitations. The present study did not perform an in-depth analysis of how AAV genetic modification alters the HGF-MSC CSs, GFP-MSC CSs secretome and vesicle/exosome profile. Such data are critical for understanding the mechanisms by which HGF modulates autophagy and other key cellular processes, including cell survival, proliferation, and death. A second major limitation is the use of a specialized in vivo cryoinjury heart model. While technically straightforward and associated with high postoperative survival rates, consistent scar formation, and epicardial cell activation, the model’s limited tissue damage and absence of transmural injury restrict its applicability for comprehensive assessments of post-infarction remodeling and systolic function. Future studies could focus on single-cell analysis to characterize populations derived from activated epicardium, as well as the long-term effects of HGF-MSC CSs on fibrosis and cardiac remodeling during the late stages of post-infarction repair.

In conclusion, HGF-MSC CSs can be regarded as a promising tool for affecting epicardial cells, with a potential mechanism involving the modulation of autophagy. The regulation of autophagy within a specific range after hypoxic damage has been demonstrated to result in increased the viability and proliferation of epicardial cells. The adaptive regulation of autophagy levels in ischemic areas presents new opportunities not only for preventing cell death associated with hypoxia but also for influencing inflammation, angiogenesis, and fibrosis. Furthermore, this approach holds promise for enhancing the overall regenerative processes in the heart.

## 4. Materials and Methods

### 4.1. Animals

This study utilized male mice purchased from the Andreevka nursery (Moscow region, Russian Federation). The animals were housed in individual cages at 22 °C and maintained on a 12 h light/dark cycle. They were fed with standard chow and water ad libitum. All in vivo studies were performed in accordance with the Code of Practice for the Housing and Care of Animals Used in Scientific Procedures, as well as with guidelines from the American Association for Laboratory Animal Science and the Institute of Experimental Cardiology. The animal procedures were approved by the Ethics Board of Institutional Animal Care and Use Committee of the National Medical Research Center of Cardiology, named after Academician E. I. Chazov (permit #LA/28.07.2023 (28 July 2023)).

### 4.2. MSC Isolation and Cultivation

Mouse MSCs were isolated from the subcutaneous adipose tissue of male C57/Bl6 mice, as previously described [77]. The isolated cells were cultured in 4.5 g/L D-glucose DMEM/F12 (Servicebio, Wuhan, China) containing 10% fetal bovine serum (FBS) (Gibco, Waltham, MA, USA) and 1% antibiotic/antimycotic solution (Servicebio, Wuhan, China). Early-passage cells were utilized for the CS assembly (P 3–4).

### 4.3. AAV Purification and MSC Transduction

HEK293T cells (ATTC) were transfected according to the AAV Helper-Free System (Stratagene, San Diego, CA, USA) protocol. The HEK293T cells that reached 80% confluency were co-transfected using a calcium phosphate co-precipitation method with pAAV-DJ (Cell Biolabs, San Diego, CA, USA), pHelper (Stratagene, San Diego, CA, USA) and pAAV-mHGF plasmids, which we previously constructed [10], based on the mouse HGF-coding sequence (NM_001289458.1). AAV purification was performed as previously described [10]. Transduction was performed on 70% confluent MSCs by replacing the media for DMEM + 3 mL of viral stock. The dish with the cells was at 37 °C and 5% CO_2_ (with tapping every 30 min). Three hours after infection, 5 mL of DMEM, containing 20% fetal bovine serum (FBS) (Gibco, Waltham, MA, USA) and 2% antibiotic solution, was added. The infected cells were cultured for 48 h prior to experiments.

### 4.4. CSs Assembly

For cell sheet formation, the MSCs were seeded at 250 × 10^6^/well in a 48-well culture plate (Corning, Corning, NY, USA) and incubated at 37 °C and 5% CO_2_ for 72 h. The CSs were detached following the approach previously described by Dergilev et al. [78]. In summary, the CSs were subjected to a washing process comprising three cycles in Versene solution, followed by incubation in the same solution at 37 °C in a 5% CO_2_ atmosphere until self-detachment occurred within a time frame of 2–5 min. Subsequently, the CSs were transferred to DMEM.

### 4.5. ELISA

The secretomes from GFP-MSC CSs and HGF-MSC CSs grown for 48 h in DMEM/F12 containing 10% FBS and penicillin/streptomycin were collected, centrifuged at 1000× *g*, frozen in liquid nitrogen, and stored at −70 °C until assayed. The amounts of HGF, FGF2, and VEGF were measured using Quantikine ELISA kits (Mouse HGF ELISA Kit (Abcam), Mouse FGF2 ELISA Kit (Cloud-Clone Corp., Wuhan, China) and mouse VEGFA ELISA Kit (Cloud-Clone Corp.)).

### 4.6. Analysis of Extracellular Matrix Proteins and Apoptosis in CSs

CSs (72 h) were fixed with 4% formaldehyde and probed with the following primary antibodies (Abs): anti-collagen 1 (Abclonal, Wuhan, China), anti-fibronectin (Cloud-Clone Corp.), or isotype-matched control immunoglobulins, followed by fluorophore-conjugated secondary Abs Alexa Fluor™ 647 (Invitrogen, Carlsbad, CA, USA). The stained cells were visualized using a Stellaris 5 confocal microscope (Leica, Wetzlar, Germany). To quantify the fluorescent intensity of staining, we used ImageJ software (version 1.54d, NIH, USA).

The LIVE/DEAD™ kit (Invitrogen™) was used to determine cell viability in the GFP-MSC and HGF-MSC CSs according to the manufacturer’s instructions.

### 4.7. RNA Isolation, Reverse Transcription and Real-Time Quantitative PCR

GFP-MSC and HGF-MSC CSs were grown for 48 h in DMEM/F12 containing 10% FBS and penicillin/streptomycin. Total RNA was isolated from the cells with RNeasy Mini Kit (QIAGEN, Germantown, MD, USA). First-strand cDNA was synthesized with random hexamer primers using a RevertAid^TM^ First Strand cDNA Synthesis Kit (ThermoFisher Scientific, Waltham, MA, USA). Real-time PCR was performed using SYBR Green intercalating dye (Eurogene, Moscow, Russian Federation) on a StepOnePlusTM Real-Time PCR System (ThermoFisher Scientific, Waltham, MA, USA). The primers used for PCR are listed in Table 1. The mRNA amounts between the probes were normalized to the mRNA quantity of ACTB (Actin Beta), a housekeeping gene, and the 2^−ΔΔCt^ method was used to calculate the relative expression levels of the genes.

### 4.8. Heart Cryoinjury Modeling and Cell Sheet Transplantation

C57Bl6 mice, 10 weeks old with a body weight of 20–22 g, underwent cryoinjury to induce acute myocardial infarction, as described previously [79]. Briefly, mice were anesthetized with Tribromoethanol (Avertin) 100 mg/kg body weight, injected intraperitoneally), and positive pressure respiration was applied through an endotracheal tube. The left thorax was opened via intercostal space, and the heart wall was damaged by a stainless rod frozen in liquid nitrogen.

The mice were randomly assigned to the following four groups: (1) sham-operated (*n* = 4), (2) Cryoinjury (*n* = 12), (3) Cryoinjury + GFP-MSC CSs (*n* = 12), and (4) Cryoinjury + HGF-MSC CSs (*n* = 12). Twenty minutes after artery ligation, the corresponding cell sheet was detached from the culture plate, untucked by sterile needle forceps, and then transferred (using a low-adhesive membrane) onto the epicardium layer (to cover the pale ischemic area). The heart was returned to the chest cavity, and the muscles and skin were closed in layers. Control animals (sham-operated and cryoinjury groups) underwent the same procedure, except for cell sheet transplantation.

### 4.9. Flow Cytometry Analysis

To determine c-Met expression, MEC-1 cells were washed twice with ice-cold PBS, resuspended in ice-cold PBS supplemented with 1% BSA, incubated with primary unlabeled anti-Met (Becton Dickenson, Franklin Lakes, NJ, USA; #560839; 1:100) antibodies for 30 min at 4 °C, washed with ice-cold PBS, incubated with secondary anti-rat-Alexa 488 antibodies for 30 min at 4 °C, and washed with ice-cold PBS and fixed in PBS containing 1% paraformaldehyde. Flow cytometry analysis was performed with a FACS Aria III (Becton Dickinson, Franklin Lakes, NJ, USA). The analysis was performed using the gating principle. First, the total cell population was isolated based on forward scatter (FSC) and side scatter (SSC). Next, doublets (cell clusters) were excluded by comparing FSC-A with FSC-H. Finally, the population of interest was quantified.

A Cell Cycle Assay Kit (Elabscience, Wuhan, China) was used for cell cycle analysis, as previously described [80,81]. Briefly, Mec-1 cells were incubated for 36 h in control or conditioned mediums (GFP-MSC CS and HGF-MSC CS). Then, the epicardial cells were detached with 0.025% Trypsin-EDTA, washed with phosphate-buffered saline (PBS), and suspended in PBS to generate a single suspension. The cells were then fixed in methanol overnight at −20 °C. The methanol was removed by centrifugation, and the EMCs were suspended in PBS containing RNase A for 30 min at 37 °C and then stained with propidium iodide. DNA fluorescence was measured on a FACS Aria III system (Becton Dickinson, USA).

The apoptosis levels in the Mec-1 after exposure to CoCl_2_ and conditioned media were analyzed by Annexin V and propidium iodide double staining according to the manufacturer’s instructions (BD Pharmingen, San Diego, CA, USA). The attached cells were harvested using trypsin-EDTA solution, combined with floating cells, washed in PBS, and resuspended in a binding buffer. Fluorescein isothiocyanate (FITC)-annexin V and PI were added, and the mixture was incubated at room temperature for 30 min before analysis. The analysis was performed by flow cytometry using BD FACS Aria III (Becton Dickinson, Franklin Lakes, NJ, USA). The analysis was performed using a gating strategy. First, the total cell population was isolated based on FSC and SSC. Next, doublets (cell clusters) were excluded by comparing FSC-A with FSC-H (or FSC-W). Finally, subpopulations were analyzed using Annexin V-FITC versus propidium iodide staining to distinguish live, apoptotic, and dead cells. Viable, apoptotic, and necrotic cells were detected, with their number determined as a percentage of the whole population.

### 4.10. Prestoblue Assay

Cell viability was tested using the Presto Blue assay, as described previously with modifications [81]. The mouse EMCs were plated in 96-well culture plates (2 × 10^3^ cells/well). The cells were incubated for 24, 48, and 72 h, and 10 µL of the fluorescent dye PrestoBlue™ Cell Viability Reagent (Invitrogen, USA) was added to the culture medium for an additional hour. The fluorescence intensity was measured using a Victor X3 spectrophotometer (PerkinElmer, Waltham, MA, USA) at a wavelength of 570 nm, with the number of cells per well determined by plotting a standard curve.

The effect of HGF and autophagy level on the survival of EMCs was investigated by chemically modulating hypoxia using CoCl_2_ (at the indicated doses) and by blocking autophagy with the inhibitor-3-methyladenine (3 MA; 10 mkM).

The c-Met inhibitor, crizotinib (Selleckchem, Houston, USA), was used in experiments to evaluate the effect of HGF-MSC CSs secretome on cell proliferation. Crizotinib was dissolved in DMSO and diluted in cell culture mediums to a working concentration of 100 nM prior to use. The DMSO content in the working solution did not exceed < 0.1%.

### 4.11. Cyto-ID Immunostaining

Cyto-ID, a special tracer dye, labels autophagy vacuoles with minimal staining of lysosomes, suggesting it is a specific marker for monitoring autophagy [82]. The Cyto-ID^®^ Green Detection Kit (Sigma, St. Louis, MO, USA) was used to visualize autophagy vesicles. Mec-1 (1 × 10^4^) was cultivated on glass chamber slides in the absence or presence of CoCl_2_ for 24 h. The sample was stained with Cyto-ID^®^ reagent for 30 min at 37 °C and analyzed by Image Exfluorer (LCI, Namyangju-si, Republic of Korea).

### 4.12. Western Blot Analysis

Western blotting was performed using specific antibodies against anti-MET (Bio-Rad, Hercules, CA, USA), -pMET (LifeSpan BioSciences, Lynnwood, WA, USA), -AKT (Cell Signalling, Boston, MA, USA), -pAKT (Cell Signalling, Boston, MA, USA), -ERK (Cell Signalling, Boston, MA, USA), -pERK (Cell Signalling, Boston, MA, USA), -Tubulin (Cell Signaling, Boston, MA, USA) and -p62/SQSTM1, -LC3, and -Atg5 produced by Abclonal (China). The EMCs were obtained by treating mouse hearts (on day 2 after cryoinjury) with a 0.25% trypsin solution (Paneco, Moscow, Russian Federation) for 5 min, followed by mechanical scraping from the heart surface, as well as Mec-1 cells (Millipore, Bedford, MA, USA) cultured in vitro under the specified conditions. The cells were lysed in the RIPA buffer containing 50 mM Tris–HCl, pH 7.4, 150 mM NaCl, 1 mM EDTA, 1% Triton X-100, 0.5% sodium deoxycholate and 0.1% SDS) supplemented with protease inhibitor cocktail (Sigma, St. Louis, MO, USA) and 1 mM phenylmethylsulfonyl fluoride (Sigma, St. Louis, MO, USA). The lysed proteins were mixed with 6× sample buffer (75 mM Tris-HCl, pH 6.8, 10% (*v*/*v*) glycerol, 2% SDS (*w*/*v*), 0.002% (*w*/*v*) bromophenol blue). In total, 20 μg of each sample was analyzed through 10% SDS-polyacrylamide gel electrophoresis and then transferred onto Immobilon-P polyvinylidene fluoride (PVDF) membranes (Merck Millipore, Bedford, MA, USA). The membranes were blocked with 5% (*w*/*v*) non-fat dry milk in Tris-buffered saline (TBS) supplemented with 0.1% Tween 20 for 1 h at room temperature and then incubated with primary antibodies at 4 °C overnight. Next, the membranes were washed and probed with secondary horseradish peroxidase (HRP)-conjugated polyclonal antibodies (Jackson ImmunoResearch, West Grove, PA, USA) for 1 h at room temperature and analyzed using an ECL Western Blotting kit (Amersham Biosciences, Piscataway, NJ, USA). The protein bands were visualized using the FusionFX gel-documenting system (Vilber Lourmat, Collégien, France) in video mode. All expression data were normalized to Tubulin. Optical density quantification was performed using Image J software version 1.54d (National Institutes of Health, USA).

### 4.13. Statistical Analysis

The data are presented as mean ± SD. Unless otherwise specified, each experiment was replicated thrice. The statistical significance of treatment differences was assessed using a Student’s *t*-test or one-way analysis of variance (ANOVA) with Tukey’s multiple comparisons test using GraphPad (Prism software (version 8), GraphPad, La Jolla, CA, USA). A *p*-value less than 0.05 indicated statistically significant results.

## Figures and Tables

**Table 1:** Primers.

Gene Name	Forward	Reversed
*Hgf*	AACAGGGGCTTTACGTTCACT	CGTCCCTTTATAGCTGCCTCC
*Vegfa*	GGAGACTCTTCGAGGAGCACTT	GGCGATTTAGCAGCAGATATAAGAA
*Fgf2*	GCGACCCACACGTCAAACTA	CCGTCCATCTTCCTTCATAGC
*Tgfb1*	TGGAGCAACATGTGGAACTC	GTCAGCAGCCGGTTACCA
*Tgfb2*	TTGTTGCCCTCCTACAGACTGG	GTAAAGAGGGCGAAGGCAGCAA
*Plau*	ATGGAAATGGTGACTCTTACCGA	TGG GCA TTG TAG GGT TTC TGA
*Plaur*	CGCCACAAACCTCTGCAAC	CTCTGTAGGATAGCGGCATTG
*Serpin1*	TGCAAAAGGTCAGGATCGAGG	ATGAAGAGGATTGTCTCTGTCGG
*Fn1*	GGAATGGACCTGCAAACCTA	GTAGGGCTTTTCCCAGGTCT
*Col1a1*	CCGCTGGTCAAGATGGTC	CTCCAGCCTTTCCAGGTTCT
*Col3a1*	CTGTAACATGGAAACTGGGGAAA	CCATAGCTGAACTGAAAACCACC
*Col4a1*	CTGGCACAAAAGGGACGAG	ACGTGGCCGAGAATTTCACC
*Actb*	GGCTGTATTCCCCTCCATCG	CCAGTTGGTAACAATGCCATGT

## Data Availability

Data are contained within the article.
